# Exosomes from adipose-derived stem cells activate sebocytes through the PI3K/AKT/SREBP-1 pathway to accelerate wound healing

**DOI:** 10.1007/s00441-024-03872-z

**Published:** 2024-02-27

**Authors:** Yingbo Zhang, Christos C. Zouboulis, Zhibo Xiao

**Affiliations:** 1 Department of Plastic and Aesthetic Surgery, The Second Affiliated Hospital of Harbin Medical University, Harbin, 150081 People’s Republic of China; 2 Departments of Dermatology, Venereology, Allergology and Immunology, Dessau Medical Center, Brandenburg Medical School Theodor Fontane and Faculty of Health Sciences Brandenburg, Dessau, Germany

**Keywords:** Exosomes, Adipose-derived stem cells, Wound healing, Sebaceous glands, PI3K/AKT

## Abstract

Sebocyte regeneration after injury is considered a key element of functional skin repair. Exosomes from adipose-derived stem cells (ADSCs-EXO) accelerate wound healing by promoting the proliferation of fibroblasts. However, the effects of ADSCs-EXO on sebocytes are largely unknown. In this study, the effects of ADSCs-EXO on sebocyte proliferation and migration were evaluated. The levels of phosphorylated AKT (p-AKT), AKT, sterol regulatory-element binding protein (SREBP), and perilipin-1 (PLIN-1) were detected with immunofluorescence, quantitative PCR, and western blot analysis. RNA-Seq was used to analyze the differential gene expression between the ADSCs-EXO group and the control group under anaerobic conditions. Lipogenesis was assessed with Nile red staining. In animal studies, full-thickness skin wounds in BALB/c mice were treated with gelatin methacrylate (GelMA) hydrogel-loaded sebocytes alone or in combination with ADSCs-EXO. Histopathological assessments of the wound tissues were performed Masson Trichrome staining, Immunohistochemical staining and so on. The phosphatidylinositol 3-kinase (PI3K)/AKT pathway blocker LY294002 inhibited the effects of ADSCs-EXO on p-AKT and sebocytes proliferation. ADSCs-EXO also regulated the expression of SREBP-1 and PLIN-1 through the PI3K/AKT pathway in an oxygen level-dependent manner. In BALB/c mice, ADSCs-EXO accelerated sebocyte-assisted wound healing and regeneration. These in vitro and in vivo results supported that ADSCs-EXO can promote the regeneration of fully functional skin after injury through the PI3K/AKT-dependent activation of sebocytes.

**Graphical abstract:**

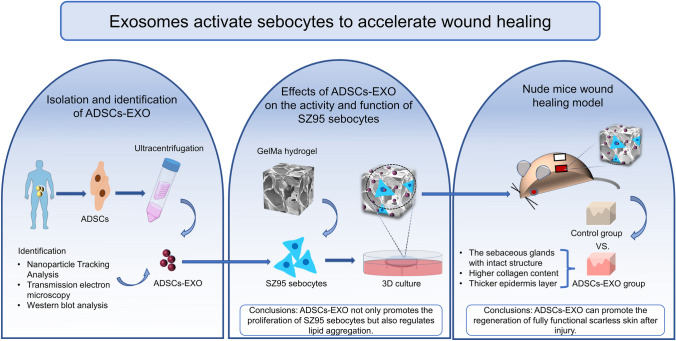

## Introduction

In healthy skin, epidermal appendages are essential for regulating body temperature, maintaining skin homeostasis, and resisting external stress (An et al. 2021; Boström et al. 2006; Brasaemle 2007). The natural healing of deep skin wounds in adult skin often involves the rapid formation of fibrotic tissues that lack the original complex skin architecture, leading to hypertrophic or atrophic scar formation (Choi et al. 2021). Compared with adjacent normal skin tissue, a significant feature of scars is the loss of sebaceous glands (SGs) (Dahlhoff et al. 2013). SGs produce and secrete an oily matter called sebum that is mainly composed of triglycerides and wax esters. Sebum lubricates the skin and protects against skin dehydration and oxidative injury (Grahn et al. 2013). SG reconstruction is necessary for the regeneration of fully functional skin (Griffin et al. 2020). In addition, hypertrophic or depressed scars resulting from natural healing affect appearance and may have a negative effect on self-esteem. Restoration of epidermal appendages including SGs is critically important for achieving scarless skin regeneration, which is highly desirable in the modern world (Griffin et al. 2021; Hu et al. 2022; Jansen et al. 2019).

Exosomes have specialized functions in physiological processes and intercellular communications, and have become a hot topic of biomedical research in recent years (Kumar et al. 2019). In particular, exosomes from adipose-derived stem cells (ADSCs-EXO) have been shown to accelerate skin wound healing by promoting the migration, proliferation, and collagen synthesis of fibroblasts and keratinocytes (Lee et al. 2018; Lee et al. 2021). However, the effects of ADSCs-EXO on sebocytes, the major cell type in SGs that produces sebum, are unknown. Sebocytes also contribute to multiple cellular and molecular events that drive skin wound repair (Jansen et al. 2019).

In this study, we evaluated the effects of human ADSCs-EXO on the migration, proliferation, and lipid metabolism of SZ95 human sebocytes in vitro. The underlying molecular mechanisms were also investigated. We further evaluated the effects of gelatin methacrylate (GelMA) hydrogel-loaded SZ95 sebocytes, alone or in combination with ADSCs-EXO, on cutaneous wound healing in nude mice. The GelMA hydrogel was used as a three-dimensional (3D) carrier for SZ95 sebocytes to simulate the natural environment of sebocytes in vivo. To the best of our knowledge, this is the first study to demonstrate the therapeutic potential of ADSCs-EXO combined with sebocytes in improving the quality of wound healing.

## Materials and methods

### Cell culture

**Fig. 1:**
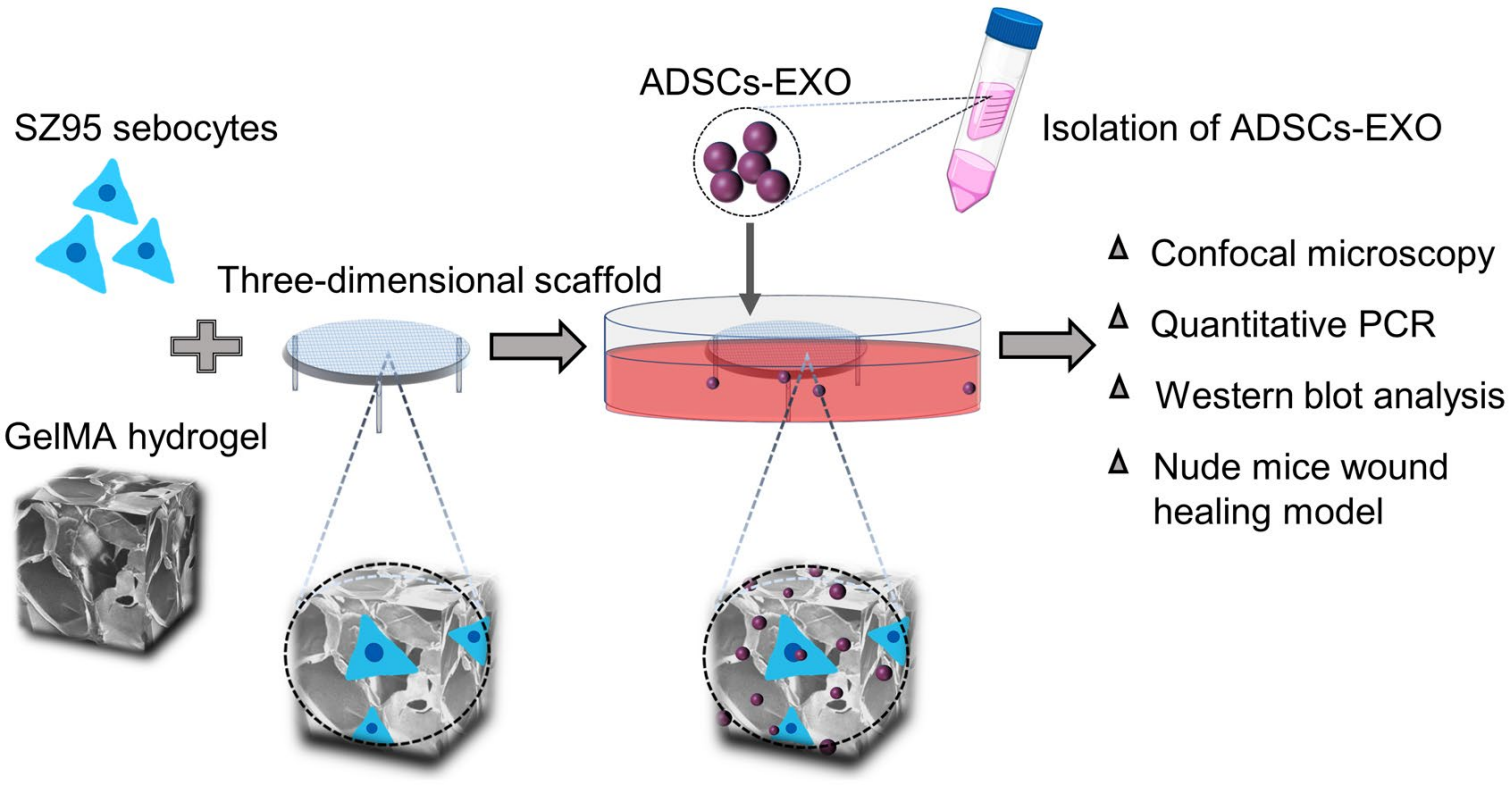
Schematic diagram depicting the preparation of GelMA hydrogel-loaded SZ95 sebocytes as an in vitro model of human SGs

The immortalized SZ95 human sebocyte cell line was maintained in Dulbecco’s modified Eagle’s medium (DMEM; Gibco, Waltham, MA, USA) supplemented with 10% fetal bovine serum (FBS; Gibco) and 1% penicillin/streptomycin (Gibco) at 37 °C in a humidified atmosphere of 5% CO_2_. The EFL-GM-60 GelMA hydrogel has an amino substitution degree of 60 ± 5%, which has been shown to promote cell adhesion and proliferation, and protect cells from injuries caused by the injection (Li et al. 2023). The operation process of 3D culture is shown in Fig. 1. With the exception of cell proliferation, migration, and lipid metabolism experiments, the 3D cell culture method was used in western blot analysis, PCR, confocal microscopy, RNA-Seq and animal experiments. The medium was changed every 2 days. Prior to each experiment, the cells were cultured in exosome-depleted FBS for 24 h to increase their sensitivity to ADSCs-EXO. To evaluate the effects of ADSCs-EXO under hypoxic conditions, the cells were treated with ADSCs-EXO in a humidified atmosphere of 1% O_2_, 5% CO_2_, and 94% N_2_ for 24 h.

### Isolation and identification of ADSCs-EXO

Adipose tissues were harvested from five female patients (age 18–40 years) with no underlying diseases, who received liposuction at The Second Affiliated Hospital of Harbin Medical University (Harbin, China) from January to December 2022. The protocol was approved by the ethics committee of The Second Affiliated Hospital of Harbin Medical University (Under the code: KY2022-221), all methods were carried out in accordance with relevant guidelines and regulations. All patients provided written informed consent. The adipose tissues were washed several times with sterile phosphate-buffered saline (PBS) and digested with collagenase type I (0.1%; Gibco) at 37 °C for 100 min. After filtering through a 100-μm filter, the filtrate was centrifuged at 800 RCF for 5 min. The resulting cell pellet was resuspended and cultured in DMEM F12 (Gibco) supplemented with 10% FBS. The cells at 2–5 passages were grown to 70–80% confluence, washed with PBS, and cultured in exosome-depleted FBS for 24 h. The cell supernatant was collected, centrifuged at 1500 RCF for 10 min to remove cell debris, and filtered through a 0.22-μm sterile filter (Sterile Micropores, Burlington, MA, USA) by centrifugation at 3200 RCF for 20 min at 4 °C. The exosomes were precipitated with ExoQuick-TC (System Biosciences, Palo Alto, CA, USA) following the manufacturer’s instructions, resuspended in PBS, and stored at -80 °C. The exosomes were identified and characterized using the Libra 120 transmission electron microscope (Zeiss, Jena, Germany) and the NanoSight LM10 nanoparticle tracking system (Malvern Panalytical Ltd., Malvern, UK). The exosome surface markers cluster of tumor susceptibility gene 101 (TSG101), 70-kDa heat shock proteins (HSP70) and CD63 were detected with western blot analysis using antibodies from Abcam (Cambridge, MA, USA).

### Cell proliferation, migration, and apoptosis

To evaluate cell proliferation, SZ95 sebocytes were seeded in 6-well plates (1 × 10^6^ cells/well), grown to 60 − 70% confluency, and treated with ADSCs-EXO (20 μL) or vehicle control overnight. Cell viability was determined with the EdU colorimetric immunohistochemistry assay (Beyotime, Shanghai, China). To evaluate cell migration with the scratch assay, the cells were seeded in 6-well plates (1 × 10^6^ cells/well), a scratch wound was created using a pipette tip, and treated with ADSCs-EXO (20 μL) or vehicle control for 24 h. The widths of the wound bed were determined after incubation for another 12 or 24 h. To evaluate cell migration with the Transwell assay, the cells were added to the upper Transwell chamber (1 × 10^6^ cells/chamber) and incubated with ADSCs-EXO (20 μL) or vehicle control for 24 h. The cells that had migrated to the lower surface of the membrane were fixed, stained with crystal violet, and counted under an inverted light microscope. To evaluate cell apoptosis, the cells (2–3 × 10^6^) were treated with ADSCs-EXO (40 μL) or vehicle control overnight. Cell apoptosis was determined with flow cytometry using the Annexin V FITC Apoptosis Detection Kit from Beyotime.

### Quantitative PCR

Total RNA was isolated using Trizol reagent. cDNA was synthesized using a reverse transcription kit (Seven Biotech, Beijing, China). The mRNA levels of sterol regulatory-element binding protein 1 (SREBP-1), perilipin-1 (PLIN-1) and AKT were determined with quantitative PCR (qPCR) using SYBR Green (Seven, China). The primer sequences used for the amplification reactions are shown in Table 1. All primers were from Sangon Biotech (Shanghai, China).

**Table 1:** Primers used in RT-qPCR analysis

Transcript	Primer(5′-3′)	Length
SREBF1	Forward: GCCCCTGTAACGACCACTG Reverse: CAGCGAGTCTGCCTTGATG	**19** **19**
AKT	Forward: AGCGACGTGGCTATTGTGAAG Reverse: GCCATCATTCTTGAGGAGGAAGT	**21** **23**
PLIN1	Forward: TGTGCAATGCCTATGAGAAGG Reverse: AGGGCGGGGATCTTTTCCT	**21** **19**
GAPDH	Forward: CTGCCCAGAACATCATCC Reverse: CAGATGCCTGCTTCAC	**18** **16**

### Western blot analysis

SZ95 sebocytes encapsulated in GelMA hydrogel were recovered by centrifugation at 800 RCF for 5 min, washed with cold PBS, and incubated in lysis buffer for 15 min. The resulting lysates were centrifuged at 3200 RCF, 4 °C for 15 min. The protein concentrations were determined using the BCA protein assay (Beyotime). The proteins were resolved by sodium dodecyl sulfate polyacrylamide gel electrophoresis, electrotransferred to 0.45-μm polyvinylidene fluoride membranes (Millipore, Mississauga, Canada), and incubated with antibodies against GAPDH (1:2000, ab181602; Abcam), phosphorylated AKT (p-Akt) (1:1500, 13038; Cell Signaling Technology, Danvers, MA, USA), AKT (1:1500, 9272; Cell Signaling Technology), SREBP-1 (1:1000, ab3259; Abcam), and PLIN-1 (1:1000, ab172907; Abcam) at room temperature for 1 h. After blocking in 5% nonfat milk for 1 h, the membranes were incubated with horseradish peroxidase-conjugated secondary antibodies at room temperature for 1 h. The immunoreactivity was detected with the ECL Detection Reagent (Beyotime). Protein bands were digitally scanned with the ImageQuant LAS 4000 mini machine (GE Healthcare, Chicago, IL, USA).

### RNA-Seq and data analysis

RNA was extracted using TRIzol Plus RNA Purification kit (Thermo Fisher Scientific, Waltham, MA, USA). RNA library preparations and sequencing reactions were conducted at GENOME (Beijing, China). For experiments in which biological replicates were set, we adopted DESeq2 for differential gene expression analysis. The main reference for differential gene screening is fold change value and q value (*p* < 0.05, |log2 fold change|≥ 1). Normalized read counts > 100 were used for heatmap visualization and further analyses.

### Cell staining and immunofluorescence

To detect the cellular or tissue uptake of ADSCs-EXO, ADSCs-EXO were labeled with PKH26 using the PKH26 Red Fluorescent Cell Linker Kit (Sigma, St. Louis, MO, USA). Lipid formation was evaluated with Nile red staining, and nuclei were stained with Hoechst 33,442. Immunofluorescence for p-AKT detection was carried out using an anti-p-AKT antibody from Cell Signaling Technology following the manufacturer’s instructions. Images were acquired sequentially on a confocal microscope (Zeiss) equipped with ZEN software. Raw images were analyzed using ImageJ software. Each experiment was repeated three times, and representative images are presented in the figures.

### Statistical analyses

All results are presented as the mean ± standard deviation (SD) of at least three independent experiments. Data were analyzed with Microsoft Excel, ImageJ, and GraphPad Prism 9. Two groups were compared using Student’s t-test (normally distributed data) or the Mann–Whitney U test (nonnormally distributed data). Analysis of variance was used for comparisons between groups of continuous variables. The *t*-test or one-way analysis of variance was used to evaluate the differences between two groups. *P* < 0.05 was considered statistically significant.

### Nude mice wound healing model

Female BALB/c nude mice (5 weeks old, 170–200 g, specific pathogen-free) were obtained from The Animal Experiment Center at The Second Affiliated Hospital of Harbin Medical University. The mice were anesthetized with intraperitoneal injection of 10% chloral hydrate solution (250 μL/100 g). Two symmetrical rounded full-thickness skin wounds with a diameter of 0.8 cm were created on the back of each mouse. GelMA hydrogel (100 μL) loaded with 1 × 10^6^ SZ95 sebocytes and 50 μL PKH26-labeled ADSCs-EXO were injected into one wound, and GelMA hydrogel (100 μL) loaded with 1 × 10^6^ SZ95 sebocytes and 50 μL PKH26 control was injected into the other wound. The wounds were photographed at 0, 1, 3, 5, 7, 9, 11, and 13 days after the injection. Wound tissues were collected 14 days after the injection. PKH26-labeled ADSCs-EXO were detected with fluorescence microscopy. Histopathological assessments of the wound tissues were performed with hematoxylin and eosin, Masson Trichrome staining, Oil red staining and Immunohistochemical staining. The study protocol was in compliance with the Institutional Animal Care and Use Committee Guidelines and was approved by the ethics committee of The Second Affiliated Hospital of Harbin Medical University (Under the code: SYDW2022-090).

## Results

### ADSCs-EXO characterization

**Fig. 2:**
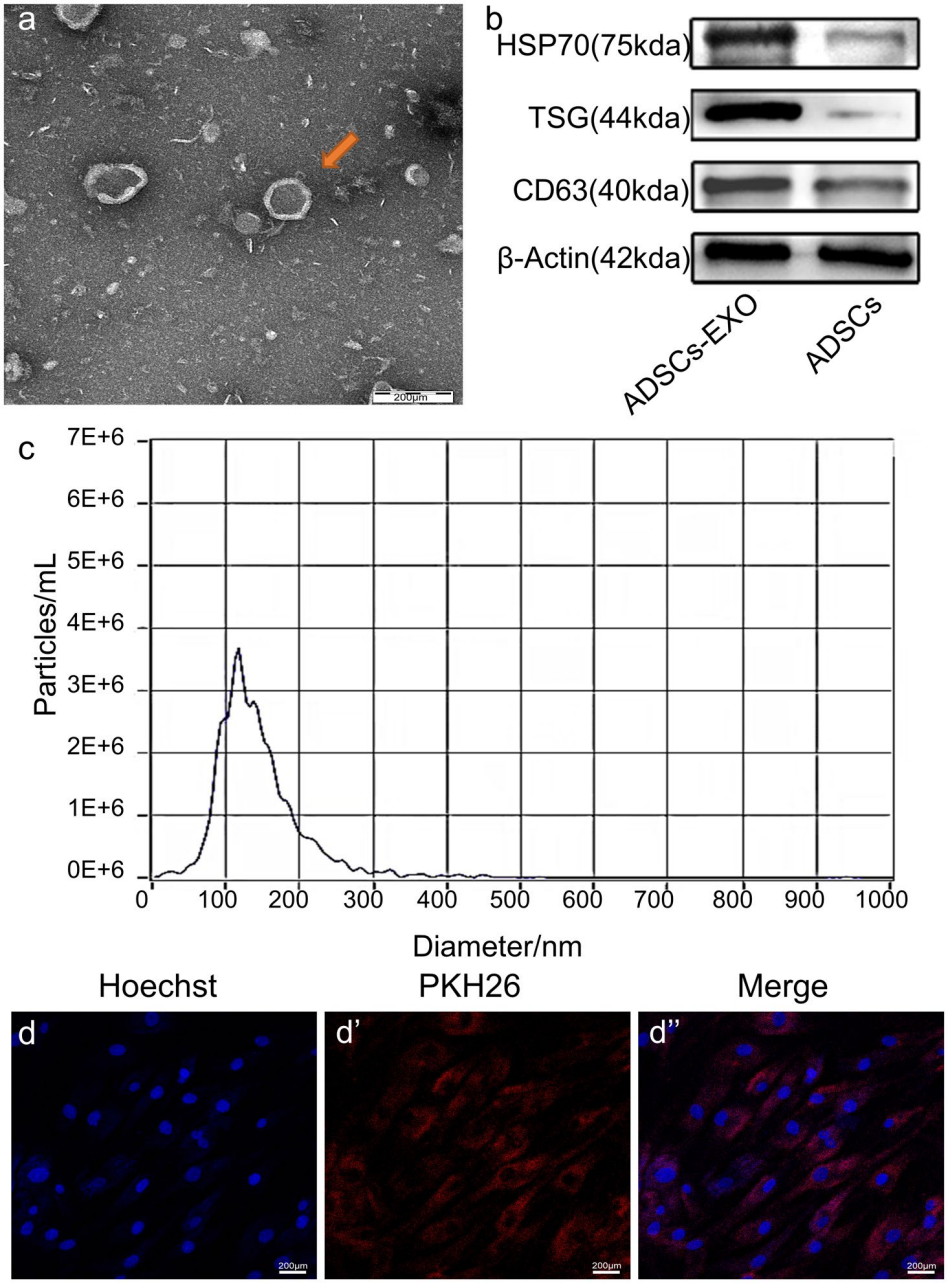
Characterization of ADSCs-EXO. **a** Representative transmission electron microscopy images of ADSCs-EXO. **b** Detection of the exosome markers, HSP70, TSG101 and CD63 in three different lots of ADSCs-EXO with western blot analysis. **c** Particle size distribution of ADSCs-EXO. **d-d**’’ Detection of PKH26-labeled ADSC-EXO in SZ95 sebocytes by fluorescence imaging. Abbreviations: ADSCs-EXO, exosomes from adipose-derived stem cells

Under transmission electron microscopy, the ADSCs-EXO appeared as sphere-shaped, bilayer vesicles with typical exosome morphology (Fig. 2a). Western blot analysis confirmed the presence of the exosome markers TSG101, HSP70, and CD63 in three different lots of ADSCs-EXO (Fig. 2b). Nanoparticle Tracking Analysis (NTA) showed that the particle size distribution of ADSCs-EXO was 117.7 nm in the peak area, the peak percentage was 96.2%, and the concentration was 2.0E + 11 particles/mL (Fig. 2c). PKH26-labeled ADSCs-EXO (red) was detected in the area surrounding the nucleus of SZ95 sebocytes by fluorescence imaging (Fig. 2d-d’’), verifying the cellular uptake of these extracellular vesicles.

### ADSCs-EXO accelerate the migration and proliferation of SZ95 sebocytes

**Fig. 3:**
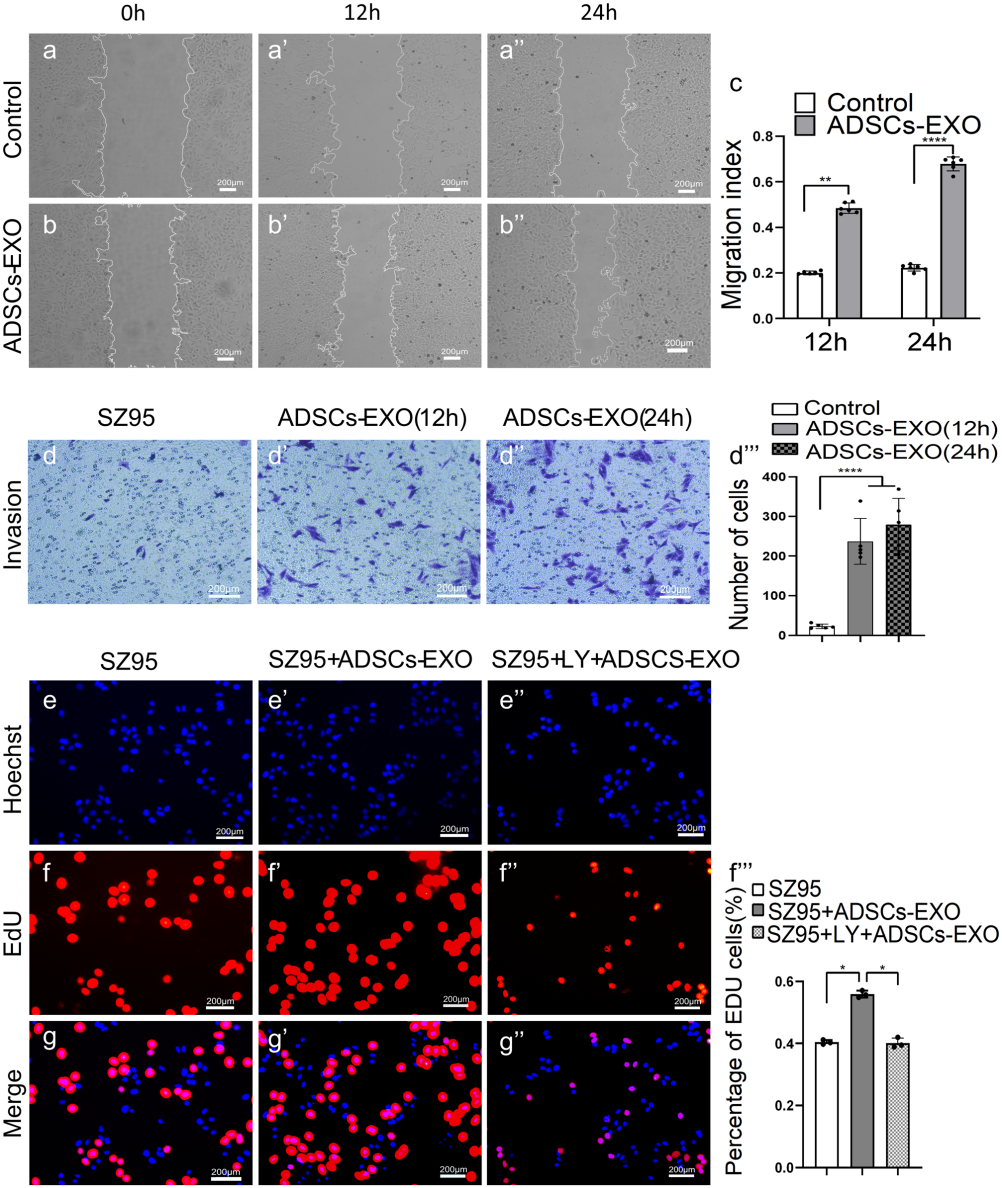
ADSCs-EXO accelerate the migration and proliferation of SZ95 sebocytes. (**) *P* < 0.01, (****) *P* < 0.0001. SZ95 sebocytes were treated with ADSCs-EXO as described in the Materials and Methods. Cell migration was evaluated with the scratch **a-c** and Transwell d-d’’’ assays. **e–g**’’ SZ95 sebocytes were pre-treated with LY294002 (10 μM) for 24 h, and then stimulated with ADSCs-EXO (20 μL) overnight. Cell proliferation was evaluated with the EdU colorimetric immunohistochemistry assay. Abbreviations: LY, LY294002. Scale bars = 200 μm

Fluorescence imaging revealed a large number of PKH26-labled ADSCs-EXO (red) in the cytoplasm of SZ95. Stimulation with ADSCs-EXO significantly increased the migration and proliferation of SZ95 sebocytes as shown by the scratch and Transwell assays (Fig. 3a-d’’’) and the EdU colorimetric immunohistochemistry assay (Fig. 3e-g’’), respectively.

### ADSCs-EXO accelerate the proliferation of SZ95 sebocytes via the phosphoinositide 3-kinase/AKT pathway

**Fig. 4:**
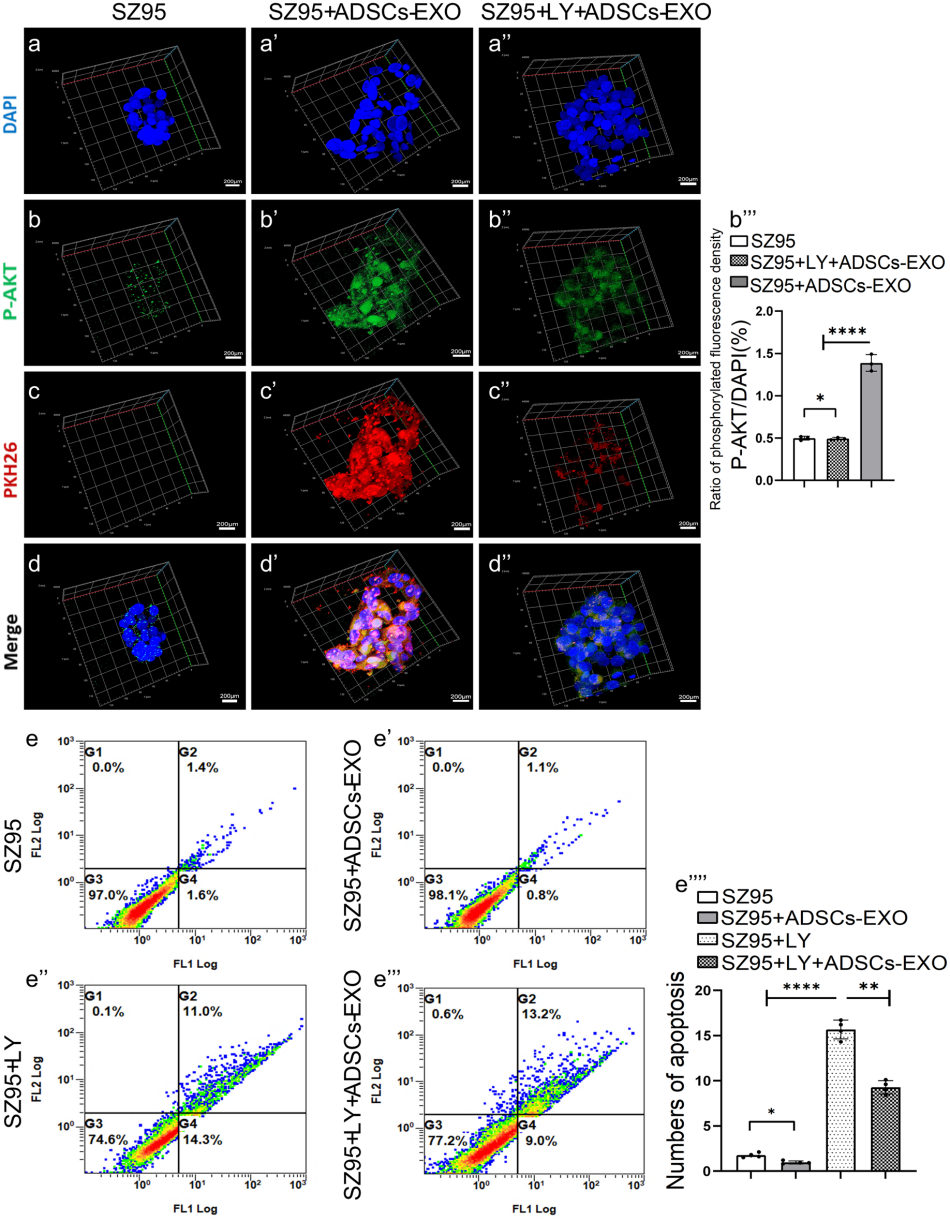
ADSCs-EXO accelerate the proliferation of SZ95 sebocytes via the PI3K/AKT pathway. (*) *P* < 0.05; (**) *P* < 0.01; (****)* P* < 0.0001. **a-d**’’ SZ95 sebocytes were pre-treated with LY294002 (10 μM) for 24 h, and then stimulated with PKH26-labeled ADSCs-EXO (20 μL) overnight. PKH26-labeled ADSCs-EXO (red) and p-AKT (green) were detected by confocal fluorescence microscopy. **e-e**’’’’ SZ95 sebocytes were pre-treated with LY294002 (10 μM) for 24 h, and then stimulated with ADSCs-EXO (20 μL) overnight. Cell apoptosis was evaluated with flow cytometry. Abbreviations: LY, LY294002. Scale bars = 200 μm

The phosphoinositide 3-kinase (PI3K)/AKT pathway is a master regulator of cell proliferation. To determine whether this pathway mediates the pro-growth function of ADSCs-EXO in SZ95 sebocytes, we tested the effects of the specific PI3K/AKT inhibitor LY294002 (MedChemExpress, Monmouth Junction, NY, USA). Based on the proliferation of cells by EdU assays, we found that ADSCs-exosome-mediated proliferation of SZ95 was attenuated by LY294002 (PI3K/AKT inhibitor) (Fig. 3e-g’’). As such, we observed PKH26-labeled exosomes in an in vitro tracking experiment via confocal microscopy. The PKH26-labeled exosomes (red) were incubated with SZ95 in basal medium with or without LY294002 (PI3K/AKT inhibitor) for 24 h. Then p-AKT was marked in green by immunofluorescence staining. We found that the addition of ADSCs-EXO increased the green fluorescence of p-AKT, and LY294002 attenuated the p-AKT fluorescence induced by ADSCs-EXO (Fig. 4a-d’’). These data support the hypothesis that ADSCs-EXO promotes the proliferation of SZ95 cells through the PI3K/AKT pathway and this effect of promoting cell proliferation is more significant in 3D culture. In addition, flow cytometry showed that the number of early apoptotic cells in SZ95 cells treated with LY294002 was significantly increased, whereas the early apoptosis of SZ95 cells treated with both LY294002 and ADSCs-EXO was controlled (Fig. 4e-e’’’’). Thus, ADSCs-EXO also protected SZ95 sebocytes from apoptosis via PI3K/AKT-dependent mechanisms.

### ADSCs-EXO regulate the lipid metabolism of SZ95 sebocytes via the PI3K/AKT pathway

**Fig. 5:**
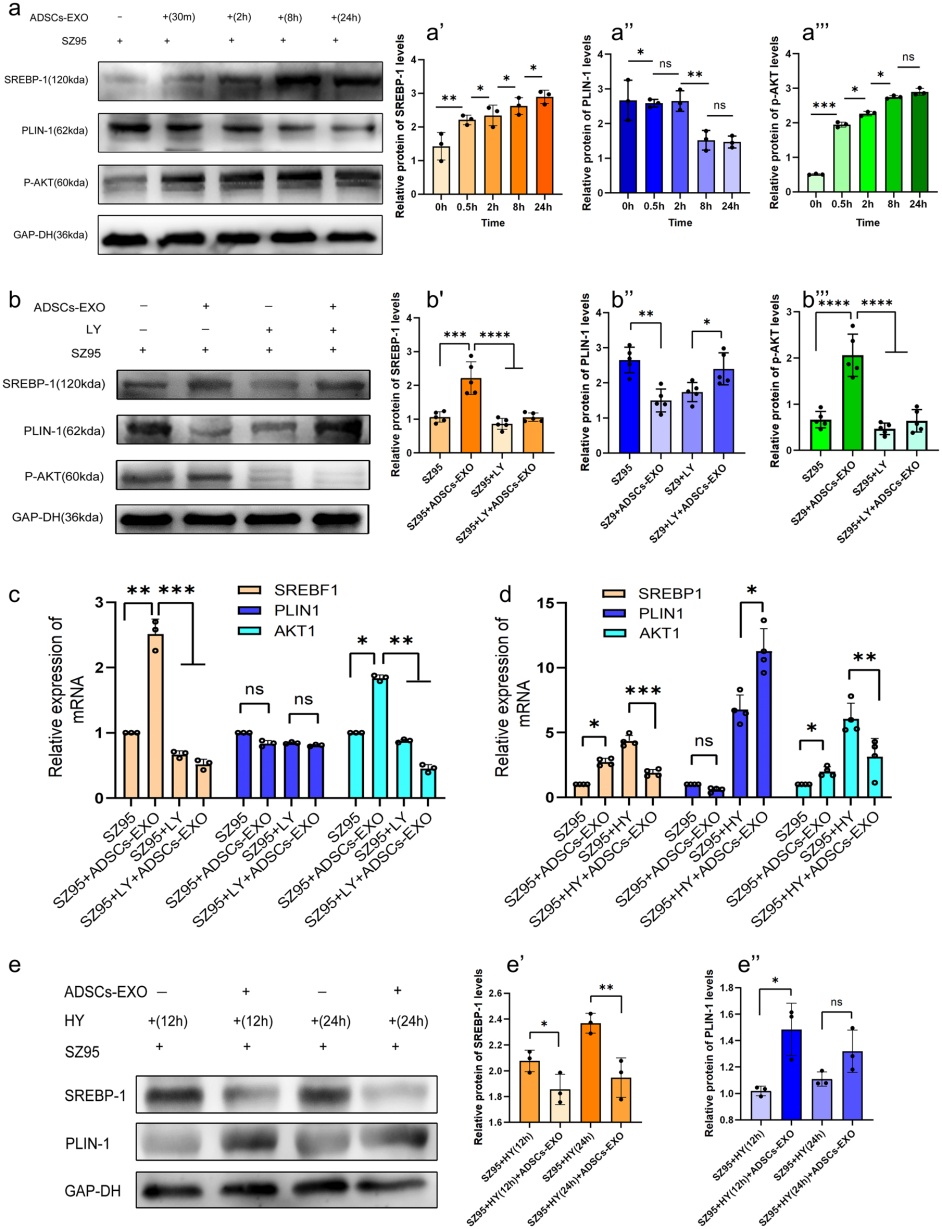
ADSCs-EXO regulate the lipid metabolism of SZ95 sebocytes. (*) *P* < 0.05, (**) *P* < 0.01, (****) *P* < 0.0001. **a-a**’’’ SZ95 sebocytes were treated with ADSCs-EXO for half-hour and 2, 8, and 24 h. The protein levels of SREBP-1, p-AKT, and PLIN-1 at different time points were detected by western blot analysis. **b-b**’’’ SZ95 sebocytes were treated with ADSCs-EXO and LY294002, alone or in combination for 24 h. The protein levels of SREBP-1, PLIN-1, and p-AKT were determined by western blot analysis. **c** SZ95 sebocytes were treated with ADSCs-EXO and LY294002, alone or in combination for 24 h. The mRNA levels of SREBP-1, PLIN-1 and AKT were determined with qPCR**. d** SZ95 sebocytes were treated with ADSCs-EXO for 24 h under hypoxic or normoxic conditions. The mRNA levels of SREBP-1, PLIN-1 and AKT were determined with qPCR. **e-e’’** SZ95 sebocytes were treated with ADSCs-EXO for 12 or 24 h under hypoxic conditions. The protein levels of SREBP-1 and PLIN-1 were determined with western blot analysis. Abbreviations: HY, hypoxic conditions; LY, LY294002; ns, no significance

SREBP-1, a transcription factor regulating lipogenesis-related genes, promotes human sebocyte proliferation and lipid synthesis (Madl et al. 2018; Mascharak et al. 2021). PLIN-1 is a protein that coats lipid droplets and regulates lipolysis. To evaluate the effects of ADSCs-EXO on lipid metabolism in SZ95 sebocytes, the mRNA and protein levels of SREBP-1 and PLIN-1 were determined with qPCR and western blot analysis, and lipid formation was assessed with Nile red staining. The results showed that ADSCs-EXO upregulated p-AKT and SREBP-1 proteins in a time-dependent manner (Fig. 5a-a’’’). Interestingly, ADSCs-EXO downregulated PLIN-1 protein after 2 ~ 8 h of stimulation, and this suppressive effect lasted up to 24 h (Fig. 5a-a’’). Mechanistically, western blot analysis showed that AKT phosphorylation, SREBP-1 upregulation, and PLIN-1 downregulation induced by ADSCs-EXO under normoxia were all reversed by LY294002 treatment (Fig. 5b-b’’’), suggesting that ADSCs-EXO regulates sebocyte lipid metabolism through the PI3K/AKT pathway. The qPCR data showed that ADSCs-EXO upregulated SREBP-1 mRNA expression, but did not significantly change PLIN-1 mRNA expression (Fig. 5c and d), suggesting that ADSCs-EXO downregulated PLIN-1 protein post-transcriptionally.

**Fig. 6:**
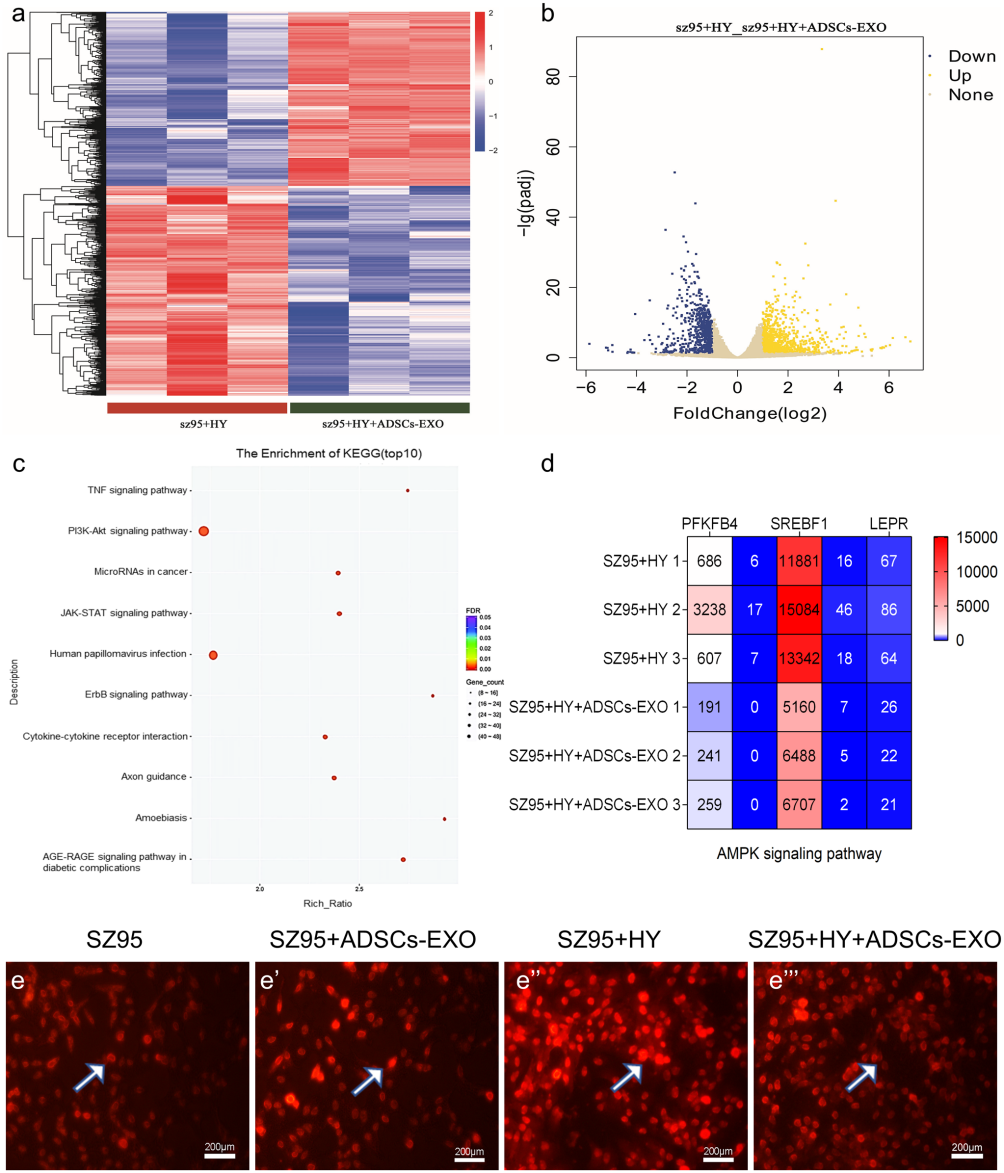
Transcriptome analysis of SZ95 + HY and SZ95 + HY + ADSC-EXO. **a** Differentially expressed genes in SZ95 + HY (left; red) and SZ95 + HY + ADSC-EXO (right; green) (N = 3, *P* < 0.05). **b** Volcano plot of differentially expressed mRNAs depicting upregulated and down egulated mRNAs. **c** Significant terms in Kyoto Encyclopedia of Genes and Genomes pathway analysis. **d** Heatmaps of differentially regulated genes involved in response to AMPK signaling pathway. **e-e**’’’ SZ95 sebocytes were treated with ADSCs-EXO for 24 h under hypoxic or normoxic conditions. Lipid formation was evaluated with Nile red staining. Abbreviations: HY, hypoxic conditions. Scale bars = 200 μm

Considering that wound tissues are often exposed to nutrient- and oxygen-poor microenvironments, we investigated whether the effects of ADSCs-EXO on SZ95 were different under hypoxic conditions. Hypoxia alone upregulated AKT, SREBP-1, and PLIN-1 mRNA expression in SZ95 sebocytes (Fig. 5d-e’’) (Mascharak et al. 2020; Mascharak et al. 2022; Raposo and Stoorvogel 2013). In contrast to their effects under normoxia, ADSCs-EXO downregulated SREBP-1 and upregulated PLIN-1 under hypoxic conditions (Fig. 5d-e’’). The SZ95 + HY (hypoxic conditions) + ADSC-EXO group served as the control group in the analysis. Transcriptome profiling identified over 1793 gene targets that were differentially expressed (Fig. 6a and b), including 980 genes that were significantly upregulated, and 813 genes that were downregulated. Kyoto Encyclopedia of Genes and Genomes pathway analysis revealed the PI3K/Akt signaling pathway as the most enriched one in Top10. (Fig. 6c). AMPK signaling pathway related gene heat map showed that the expression level of SREBP-1 was significantly decreased in the SZ95 + HY + ADSC-EXO group compared with SZ95 + HY group (Fig. 6d). Nile red staining revealed that hypoxia alone increased lipid accumulation (Fig. 6e and e’’), in accordance with previously reported lipogenic effects of hypoxia in multiple cell types including SZ95 sebocytes. However, consistent with the RNA-Seq results, ADSCs-EXO triggered lipolysis under hypoxic conditions. (Fig. 6e-e’’’). In conclusion, ADSCs-EXO regulates lipid metabolism in SZ95 cells through the PI3K/AKT pathway, and this regulatory process is affected by environmental oxygen content.

### ADSCs-EXO in combination with SZ95 sebocytes promote rapid, scarless would healing in BALB/c mice

**Fig. 7:**
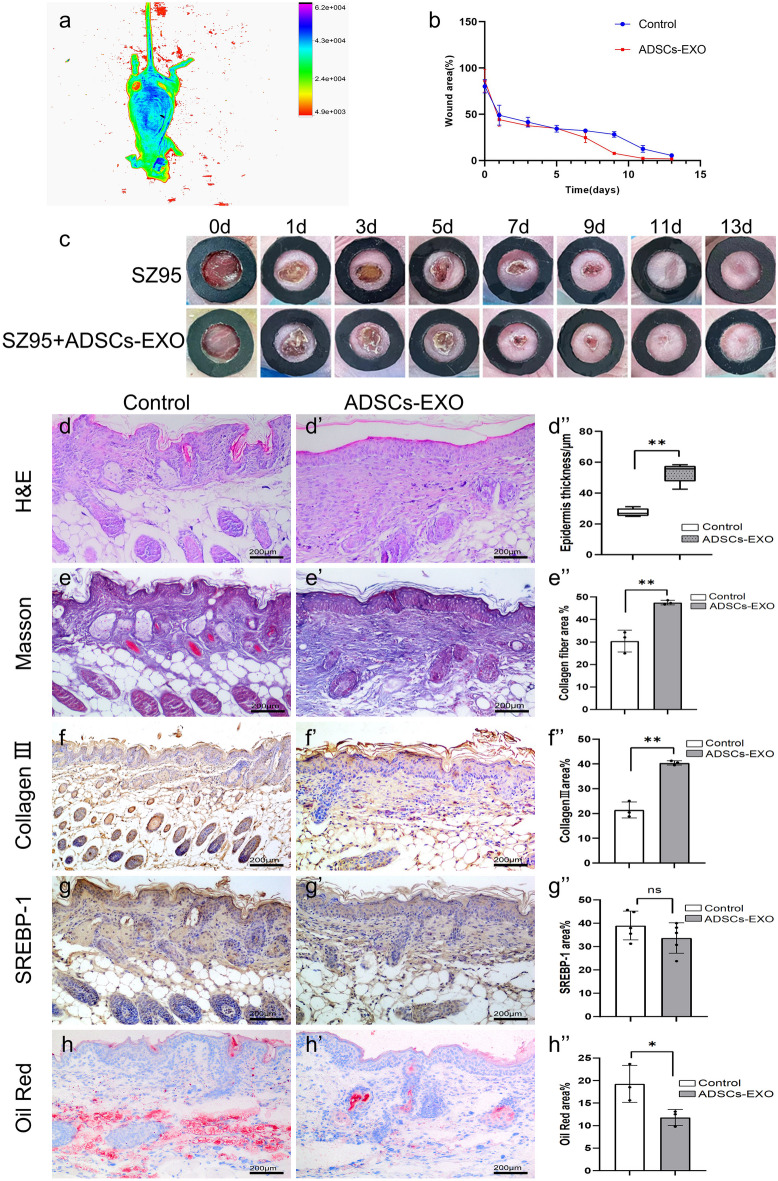
ADSCs-EXO in combination with SZ95 sebocytes promote rapid, scarless would healing in BALB/c mice. (*) *P* < 0.05; (**) *P* < 0.01. Female BALB/c nude mice with full-thickness skin wounds were treated with GelMA hydrogel loaded with SZ95 sebocytes, alone or in combination with ADSCs-EXO as described in the Materials and Methods. **a** Detection of PKH26-labeled ADSCs-EXO (red) with fluorescence imaging. **b** A chart showing the rate of wound healing. **c** Representative wound photos taken on day 0, 1, 3, 5, 7, 9, 11 and 13 post-treatment. **d-d**’’ HE staining of wound tissues on day 14 post-treatment showing epidermal thickness. **e-e**’’ Masson Trichrome staining on day 14 post-treatment showing the collagen fiber areas. **f-f**’’ Immunohistochemical staining for collagen III on day 14 post-treatment. **g-g**’’ Immunohistochemical staining for SREBP-1 on day 14 post-treatment. **h–h**’’ Oil red staining on day 14 post-treatment showing lipid accumulation areas. Abbreviations: ns, no significance. Scale bars = 200 μm

To evaluate the effects of ADSCs-EXO on sebocyte-assisted wound healing in vivo, we established a full-thickness wound healing model in female BALB/c nude mice. GelMA hydrogel loaded with SZ95 sebocytes, alone or in combination with ADSCs-EXO was injected into the wound bed. The hydrogel limited the diffusion of its payloads, leading to sustained high action concentrations of ADSCs-EXO and sebocytes at the wound bed. Fluorescence imaging verified the effective delivery of PKH26-labeled ADSCs-EXO to the injection site (Fig. 7a). The wound photos taken on days 0, 1, 3, 5, 7, 9, 11, and 13 post-treatment revealed more rapid healing of the ADSCs-EXO-treated wounds compared with control, starting from day 7 (Fig. 7b and c). On day 14, H&E and Masson staining of ADSCs-EXO-treated wound tissues showed a more organized epithelium, thicker epidermis layer, and higher collagen fiber content (Fig. 7d-e’’). Immunohistochemical staining for collagen III revealed a higher collagen content in the ADSCs-EXO-treated wounds than control (Fig. 7f-f’’). Of note, immunohistochemical staining for SREBP-1 indicated that SREBP-1 was mostly localized to the epidermis where the SGs usually reside (Fig. 7g-g’’). Moreover, more SGs with an intact structure were observed in the ADSCs-EXO group. Although the total SREBP-1-positive area in the ADSCs-EXO group was not statistically different from the control, SREBP-1 immunoreactivity in the ADSCs-EXO group appeared to decrease with increasing depth of the regenerated skin (Fig. 7g-g’’), in accordance with the suppressive effects of ADSCs-EXO on SREBP-1 under hypoxic conditions. Because of reduced lipid accumulation in the deep layer of the epidermis, the ADSCs-EXO group had a lower total lipid content than control (Fig. 7h-h’’). This could be explained by lipolysis induced by ADSCs-EXO under hypoxic conditions of deep skin. The ADSCs-EXO-induced lipolysis and consequent lipid secretion would promote the production of sebum that lubricate and protect the new skin, making it stronger (Romani et al. 2019).

## Discussion

In higher mammals, rapid formation of fibrous tissues to close skin wounds and restore the barrier function of the skin may have an evolutionary advantage. However, excessive scarring caused by disorganized fibrous tissue formation has profound psychological and physical effects on individuals, significantly reducing their quality of life after disease (Schneider et al. 2010; Scorletti et al. 2021). How to achieve rapid wound healing without compromising skin appearance and function remains a difficult medical challenge for clinicians and researchers.

Previous studies have shown that ADSCs may play a major biological role by releasing exosomes with paracrine signaling functions. Compared with ADSCs, ADSCs-EXO have more stable biological activity and lower antigenicity, and are easier to preserve and transport (Shimano et al. 2017). By alleviating inflammation, stimulating vascularization, and enhancing the proliferation and migration of keratinocytes, fibroblasts, and epithelial cells, ADSCs-EXO can accelerate skin wound healing (Smith et al. 2006) and therefore, are considered a promising therapy for the treatment of cutaneous wounds. However, ADSCs-EXO alone cannot restore the epidermal appendages that are critical constituents of fully functional skin (Schneider et al. 2010; Sun et al. 2014). To date, the effects of ADSCs-EXO on SG cells have not been explored, and the interaction between ADSCs-EXO and SG cells during wound regeneration remains unknown. SG cells can form heterogeneous structures and complete their secretion function in vivo, but this process is difficult to achieve in 2D culture. To better simulate the in vivo environment, we selected the GelMA hydrogel as a 3D environment for SZ95 sebocytes in this study to explore the effects of ADSCs-EXO on SZ95 SG cells. We found that human ADSCs-EXO enhanced proliferation and migration, and inhibited the apoptosis of SZ95 sebocytes through the PI3K/AKT pathway.

Mature differentiated SG cells secrete sebum through the accumulation of lipid droplets (Sun et al. 2022; Tachi et al. 2008). Sebum not only contributes to the integrity of the skin barrier but also helps transport antioxidants to the skin surface and exhibits antimicrobial activity (Walmsley et al. 2015). The high expression of SREBP-1, a transcription factor that regulates lipid formation, is directly related to the proliferation of SG cells and SG adipogenesis (Wang et al. 2017). Human keloids have lower levels of SREBP-regulated genes as well as diminished lipid and cholesterol-ester contents (Wang et al. 2016; Weng et al. 2020). These findings implicate SREBP-1 as an important player in wound healing and scar repair. The PLIN family of lipid droplet-associated proteins (PLIN-1 to PLIN1-5) is expressed in human sebocytes and controls sebaceous lipid accumulation (Yang et al. 2020). Under basal conditions, PLIN-1 protects lipid droplets from the body’s natural lipases (Zouboulis CC 2004). In cases of energy deficiency, PLIN-1 is hyperphosphorylated by protein kinase A, exposing the lipid droplets to lipolysis by hormone-sensitive lipases (Zouboulis et al. 1999). In this study, ADSCs-EXO regulated SREBP-1 and PLIN-1 in SZ95 sebocytes via PI3K/AKT-dependent mechanisms in an environment-dependent manner. Consequently, ADSCs-EXO enhanced lipogenesis under normoxia but triggered lipolysis under hypoxia. Considering that wound tissues are often exposed to nutrient- and oxygen-poor microenvironments, these findings will inform the potential clinical application of ADSCs-EXO in combination with sebocytes for treating cutaneous wounds under various conditions (e.g., wound depth, location, and oxygen status). In BALB/c nude mice, GelMA hydrogel loaded with ADSCs-EXO and SZ95 sebocytes achieved rapid, complete, and scarless wound healing. Histopathological examination showed that the tissue structure in the treated group was significantly different from that in the untreated control group after wound repair. In the experimental group, the epidermis was arranged in order, the epidermis was thicker, the content of type III collagen was higher, the deep lipid deposition was less, and some SG-like structures were formed, suggesting that the regenerated skin in the treated group had higher functionality than control. These in vitro and in vivo results showed that ADSCs-EXO can promote the regeneration of fully functional scarless skin after injury through PI3K/AKT-dependent activation of sebocytes. To the best of our knowledge, this is the first study to report that ADSCs-EXO can improve the speed and state of skin wound healing by activating sebocytes. Whether ADSCs-EXO can further interact with activated sebocytes through paracrine signaling on wound healing remains to be investigated. The current findings will serve as the basis for future research on functional skin regeneration and wound repair.

## Conclusions

In conclusion, treatment with ADSCs-EXO in combination with sebocytes achieved rapid and high-quality skin wound healing. This approach may showcase a therapeutic strategy for the restoration of epidermal appendages and regeneration of fully functional skin.

## Data Availability

The datasets are not publicly available due to restrictions used under the license for the current study. There are available on reasonable request from the corresponding author.

## Abbreviations

SGs: Sebaceous glands
ADSCs-EXO: Exosomes from adipose-derived stem cells
GelMA: Gelatin methacrylate
PBS: Phosphate-buffered saline
SREBP: Sterol regulatory-element binding protein
PLIN-1: Perilipin-1
LY: LY294002
HY: Hypoxic conditions
TEM: Transmission electron microscopy
p-AKT: Phosphorylated AKT
qPCR: Quantitative polymerase chain reaction
SD: Standard deviation
